# Protocol to Induce the Temporary Opening of the Blood–Brain Barrier with Short-Time Focused Ultrasound in Rats

**DOI:** 10.3390/pharmaceutics15122733

**Published:** 2023-12-06

**Authors:** Jorge A. Rodríguez, Mario I. Gutiérrez, Arturo Vera, Daniel A. Hernández, Juan M. Gutiérrez, Daniel Martínez-Fong, Lorenzo Leija

**Affiliations:** 1 Bioelectronics Section, Electrical Engineering Department, Center for Research and Advanced Studies of the National Polytechnic Institute, Mexico City 07360, Mexico; jorge.rodriguezr@cinvestav.mx (J.A.R.); daniel901205@gmail.com (D.A.H.); mgutierrez@cinvestav.mx (J.M.G.); lleija@cinvestav.mx (L.L.); 2 Subdirección de Investigación Tecnológica, Consejo Nacional de Humanidades, Ciencias y Tecnologías-Instituto Nacional de Rehabilitación LGII, División de Investigación en Ingeniería Médica, Mexico City 14389, Mexico; m.ibrahin.gutierrez@gmail.com; 3 Departamento de Fisiología, Biofísica y Neurociencias, Programa de Nanociencias y Nanotecnología, Center for Research and Advanced Studies of the National Polytechnic Institute, Mexico City 07360, Mexico; daniel.martinezfong@cinvestav.mx

**Keywords:** Blood–Brain Barrier, craniotomy, focused ultrasound, reversible opening

## Abstract

Brain neurodegenerative diseases are central nervous system (CNS) affections typically common in older adults. A new therapeutic approach for them consists of providing specific drugs to the CNS through blood circulation; however, the Blood–Brain Barrier (BBB) prevents almost 100% of neurotherapeutics from reaching the brain. There are indications that Focused Ultrasound (FUS), temporarily placed in the BBB, can achieve a controlled increase in temperature at its focus, allowing temporary, localized, and reversible opening of this barrier, which facilitates the temporary delivery of specific drugs. This work presents a FUS-based protocol for the local, temporary, and reversible opening of the BBB in Wistar rats. The proposed protocol specifies certain power, treatment times, and duty cycle to controllably increase the temperature at the region of interest, i.e., the substantia nigra. Numerical simulations using commercial software based on the finite element method were carried out to determine the optimal size of the craniotomies for nearly full-acoustic transmission. Experiments in rats were performed with the parameters used during computational simulations to determine the adequate opening of the BBB. For this, craniotomies of different sizes were made at coordinates of the substantia nigra, and FUS was applied from the exterior. The opening of the BBB was evaluated using Evans Blue (EB) as an indicator of the crossing of the dye from the blood vessels to brain tissue. Numerical simulations demonstrated a major distance reached by the ultrasound focus with a bigger diameter. Experimental results show the local, temporary, and reversible opening of the BBB through a 10 mm diameter craniotomy, which effectively allowed placing the ultrasound focus over the substantia nigra, unlike a 6 mm diameter craniotomy in which there is a deviation of the focus through that window. Moreover, from these results, it was also determined that the disruption of the BBB was reversible, with an opening duration of 6 h after FUS application. The experimental work developed in this study resulted in a minimally invasive method for the temporary opening of the BBB.

## 1. Introduction

Different treatments have been recently presented in medicine for the fight against Parkinson’s disease (PD), the second most common neurodegenerative disease worldwide [1]. Patients with PD normally are people affected at the age of 60 years and older [2], with the main symptoms related to mental disorders and sensory disturbances, which affect their quality of life [3]. To fight this disease, surgical procedures, such as deep brain stimulation and pallidotomies, have been applied to patients in advanced stages of the disease [4]; however, these procedures do not stop the dopaminergic neurodegeneration nor promote neural regeneration. Some research groups have developed neurotensin vectors for neurotrophic gene delivery in PD therapies [5]; nevertheless, the delivery of therapeutic vectors through blood circulation to the Central Nervous System (CNS) is hindered by the Blood–Brain Barrier (BBB), reducing their overall effectiveness [6].

The temporal and reversive opening of the BBB would permit less invasive and more effective drug-based therapies for neurological disorders such as PD [6]. The tight junction of endothelial cells that form the BBB impedes the passage of nearly 100% of molecules to the brain, including neurotherapeutic drugs [6,7,8]. Invasive techniques, such as a direct injection in the brain, provide the drugs to the target site, bypassing the BBB, but these invasive techniques are capable of producing irreversible damage to the brain and patient [9,10]. The use of ultrasound (US) in support of PD therapies could nanodrugs or viral vectors to cross through the temporarily opening BBB without considerable damage to the brain.

Recently, focused ultrasound (FUS) has been presented as a procedure capable of achieving a noninvasive and targeted BBB disruption [11]. FUS techniques can allow the opening of the BBB locally and reversibly [12,13,14,15], causing minor damage to the surrounding tissues [16,17,18]. The efficient disruption may be influenced by variables such as work frequency [19,20], pulse repetition [21,22,23], time application [24,25], and duty cycles [26]. Experimental work has been conducted on mice [27], which has led to positive results on controlled and quantifiable tissue damage [13]. In addition to the changes in the variables in the FUS, the use of ultrasonic contrast agents, such as microbubbles [28], has been presented as an enhancer of the disruption of the BBB process [29,30,31,32,33].

Other techniques implemented to facilitate BBB disruption by ultrasound are craniotomies [34]. Investigation groups have quantified the damage after the craniotomy procedure in animals [35,36,37] and concluded that it is a safe technique to be used to avoid the attenuation effect by the skull [27,38]. The presence of the skull leads to an increase in the temperature in soft tissue; the controlled temperature increases reported do not produce ablation in the tissue [39,40,41], but provide enough energy to generate the BBB opening with low-level damage in the tissue.

In this work, a method is presented for the temporary opening of the BBB with 2 MHz focused ultrasound through craniotomies in Wistar male rats. We obtained the parameters for the protocol by performing a computational analysis by the Finite Element Method to determine craniotomy dimensions. Ultrasound focalization was effectively produced at the substantia nigra, which caused the reversible opening of the BBB after 2.5 min of ultrasound exposure. The BBB remains open for 6 h after ultrasound application with verified closure to its original and natural state.

## 2. Materials and Methods

### 2.1. Experimental Setup

**Figure 1:**
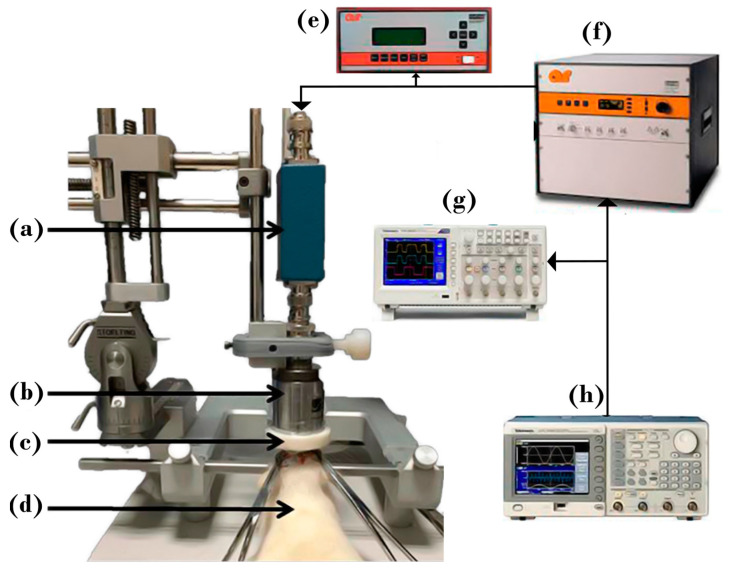
Experimental setup for FUS application in rats. Rats were anesthetized and placed on a stereotaxic system. The experimental setup comprises (**a**) the impedance coupling, (**b**) the FUS transducer, (**c**) the cone, (**d**) the Wistar male rat, (**e**) the power meter, (**f**) the power amplifier, (**g**) the digital oscilloscope, and (**h**) the signal generator.

A single concave 2 MHz transducer with a 20 mm focal length, and 20 mm diameter (Onda Corporation, Sunnyvale, CA, USA) was driven by a wave generator (6200-601841, Rohde & Schwarz, Munich, Germany) and a power amplifier (500A250, Amplifier Research, Souderton, PA, USA). A power meter (PM2002, Amplifier Research, Souderton, PA, USA) was used to measure the electrical power that was supplied to the transducer. To facilitate adequate ultrasound transmission without requiring immersion of the rat in the water, a degassed homemade thermoplastic polyurethane (TPU) cone filled with water was used as an intermediate coupler between the transducer and the rat head (Figure 1).

The transducer was characterized by a broadband hydrophone (HNP–1000, Onda Corporation, Sunnyvale, CA, USA) moved by a 3D automatic positioning system (SEA, Sunnyvale, CA, USA) in a degassed water-filled tank. The hydrophone signal was conditioned by a preamplifier (AH–2010, Onda Corporation, Sunnyvale, CA, USA); peak-to-peak and RMS voltages were recorded with an oscilloscope (TDS-420, Tektronix, Beaverton, OR, USA) and a PC using the software Scan 3.40 (Onda Corporation, Sunnyvale, CA, USA).

The transducer was driven with a sine signal of 1.965 MHz (measured frequency of operation) and an electrical power of 5 W, modulated with a tone burst of 10 Hz; the duty cycle (DC) of the FUS was controlled according to the next sequence:

1. 10 s, DC = 60%,
2. 50 s, DC = 20%,
3. 30 s, DC = 0% (rest),
4. repeat steps 1 and 2.

This protocol, which is included and detailed in the supplementary materials, allows only small temperature increases in the rat’s head, reducing the possibility of damaging the treated zone.

### 2.2. Animal Preparation

All the animals used during the experimental work were regulated by the NOM-062-ZOO-1999 for animal handling standards, and by the committee in the institution, the CICUAL (Internal Committee for the Care and Use of Laboratory Animals), with protocol 162-15 approved on 9 June 2019. The detailed protocol is included in the supplementary materials. A total of 30 male Wistar rats with weights ranging from 210 g to 232 g were used during the in vivo experiments. The total animals were divided into 10 groups for every procedure. The anesthesia solution was a composition of Xylazine (PISA agropecuaria, Nuevo México, Jalisco, Mexico) and Ketamine (PISA agropecuaria, Nuevo México, Jalisco, Mexico). This solution was intraperitoneally injected in a relationship of 32 µL for each 220 g of body weight. Once the animal was unconscious, the hair on top of the head was removed using an electric razor (Clipper Pet, Wahl, Sterling, IL, USA).

Once the rat was anesthetized, it was placed on a stereotaxic system (51600, Stoelting, Wood Dale, IL, USA). The animal was fixed at the stereotaxic system by the auditive channels with the lateral bars to maintain symmetry in the position and by the frontal part with the upper incisor teeth to immobilize the head. Then, 0.2 mL of lidocaine was subcutaneously injected to reduce bleeding when an incision of 2 cm was made on the head skin. The periosteum of the skull was removed with hydrogen peroxide to visualize the lambdoid suture, which was used as a reference. The transducer was placed 2 mm (to the left) in the transverse axis and 2.1 mm (towards the face) in the anteroposterior axis to direct the ultrasound focus to the substantia nigra.

To avoid attenuation effects produced in the rat’s skull, different square-shaped craniotomies were made using a drill (395 model, Dremel, Racine, WI, USA) and 1 mm bits. The center of the craniotomies was taken from the center position coordinate of the transducer center in the desired zone. From this point, the portion of bone to be removed was marked, taken away, and temporarily placed in a saline solution.

Before the FUS application, 1 mL of Evans blue (EB) (Molecular Weight 960.8 g/mol) [42] was injected by the dorsal vein of the tail to identify the opening of the BBB. The use of this dye was chosen because when it is administrated intravenously in the rat, the tissues are colored except the tissues in the brain due to the molecular size is not small enough to cross the BBB and its ability to join to the albumin proteins in the bloodstream [43]. After the FUS application, the removed skull piece was replaced in its original site and fixed with bone wax to avoid mobility during the animal recovery. Finally, the surgical incision was sutured, and the animal was kept in observation until its full recovery.

#### BBB Opening Detection and Reversibility Analysis

Once 24 h had elapsed after FUS application, the rat was deeply anesthetized with a dose of 0.7 mL/220 g body weight of pentobarbital (PISA Agropecuaria, Jalisco, Mexico). After the rat fell unconscious, it was euthanized with a guillotine (Nemi Scientific, Medway, MA, USA). The rat brain was carefully removed and placed in a cold saline solution to maintain the consistency of the tissue. After two minutes, the brain was placed in the base of a vibratome (VT1200S, Leica Biosystems, Wetzlar, Germany) to cut 200 µm thick slices of the encephalon with a speed of 1.5 mm/s. This procedure allowed the demonstration of EB extravasation to the brain because of the BBB opening and the precise positioning of the transducer focus at the substantia nigra.

Verification of the barrier closing and returning to the normal state was performed several hours after the FUS application to demonstrate the reversibility of its effect on the BBB. For this purpose, the ultrasound sonication was performed as explained above. Then, an EB injection was performed over time (0, 1, 6, 8, and 12 h) in independent rats after the FUS application. All the animals received the same acoustic radiation using the standard procedure described above. Each rat of the respective time point curve was submitted to euthanasia at 24 h after the EB injection to allow the dye to circulate in the bloodstream. Then, the brain was cut and observed as described above.

### 2.3. Numerical Simulation

The use of computational models can provide insights into the behavior of acoustic waves and their thermal effects on the brain for proposing optimal experimental parameters in animal studies. Therefore, acoustical and thermal approaches with the finite element method (FEM) were carried out using COMSOL Multiphysics 5.3(COMSOL Inc., Stockholm, Sweden) on a workstation with 64-GB RAM and 3.00-GHz 4-core processor (Xeon X5472, Dell, Round Rock, TX, USA).

**Figure 2:**
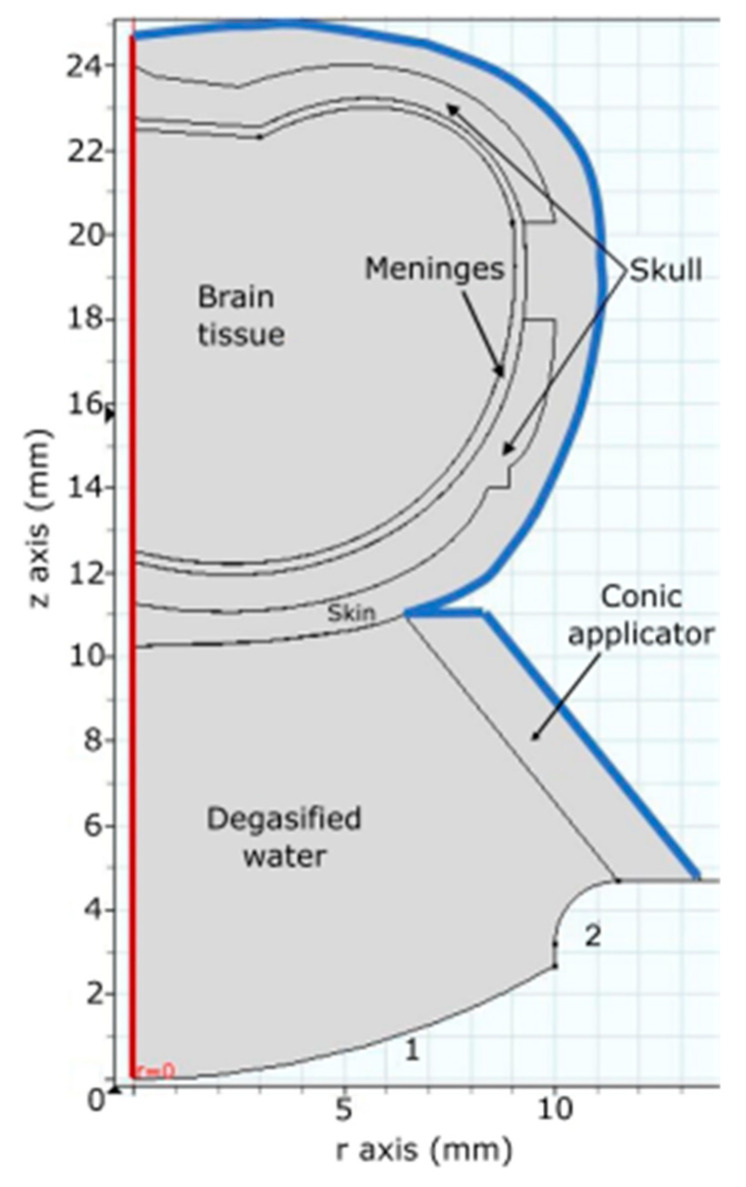
Axisymmetric geometry used for obtaining the acoustic field. Boundary 1 represents the surface of the piezoelectric, and 2 is the transducer’s case. The red boundary is the symmetry axis, the blue contour represents an impedance boundary.

The system to be modeled by FEM is symmetric along its central axis; hence the geometry employed was developed in a 2D axisymmetric model for simplification. The dimensions of thickness and curves from the rat skull brain tissue, and meninges were added to the geometry of the model (Figure 2). All internal boundaries were set with a continuous condition. Acoustic properties of the tissues of the rat head were set according to Table 1.

#### 2.3.1. Acoustic Field Distribution

Studies in the frequency domain allowed us to calculate the behavior of linear systems to harmonic excitation in one or several frequencies. For this type of study, the homogeneous Helmholtz wave equation was used, given by

(1) $$\nabla^{2}p+k^{2}p=0,$$

where p is the acoustic pressure [Pa], and k is the wavenumber [m^−1^].

With the simplification of the model, taking the entire model as a cylinder, the boundary conditions were established in the blue line of Figure 2 as an acoustic impedance for soft and hard materials added. The boundary with the number 1 was assigned with a normal acceleration that specifies the acceleration to work as an external source. The red boundary represents the geometry axis of the model.

The mesh used in the model consists of triangular elements due to the model presenting curve contours, with 9 elements per wavelength [44,45]. Mesh convergence was verified in the precision of the results obtained from simulations with an increased number of elements per wavelength, obtaining an error of less than 2%. The mechanical, acoustic, and thermal properties of the materials added to the model are described in Table 1 [46,47].

**Table 1:** Mechanical, acoustic, and thermal properties of materials and tissues [46,47].

Material	Speed of Sound c (m/s)	Attenuation α (Np/m)	Thermal Capacity, $C_{b}$ (J/kg·K)	Thermal Conductivity, k, (W/m·K)	Density, ρ (kg/m^3^)
Water	1500	0	-	-	997
Polyurethane	1900	-	-	-	35
Skin	1624	43.96	3391	0.37	1109
Skull bone	2814	113.36	1313	0.32	1908
Meninges	1545	34.18	2372	0.39	1027
Brain	1546	17.60	3696	0.49	1046

#### 2.3.2. Bioheat Transfer

The heating pattern was determined with Pennes’ bioheat transfer equation given by

(2) $$\rho C_{p}\frac{\partial T}{\partial t}-\hat{k}\nabla^{2}T=Q,$$

where ρ is the density, $C_{p}$ is the thermal capacity at constant pressure, $\hat{k}$ is the thermal conductivity, T is the temperature, and Q is the external heat source. In this case, Q is given by the ultrasonic absorption calculated by

(3) Q=2αI,

where α is the acoustic absorption coefficient [α], and I is the acoustic intensity [W/m^2^].

The study was performed in the time domain. It was set to heat during 2 min, with 1 s increments of data acquisition. Sine-wave acoustic pressure at the transducer surface was set to 200 kPa, equivalent to 5 W of electrical power and 80% of efficiency in ultrasound emission for this transducer; this would correspond to the worst-case scenario with a full wave emission (100% DC). The objective was to observe the heat pattern produced by the interaction between the ultrasonic waves and the soft tissue versus time. To analyze the craniotomy effect of skull attenuation and from other tissues, the domains that represent the meninges and the nearest part of the skull to the transducer were modified, emulating different dimensions of craniotomies.

## 3. Results

### 3.1. Modeling Acoustic Field and Temperature Distribution

**Figure 3:**
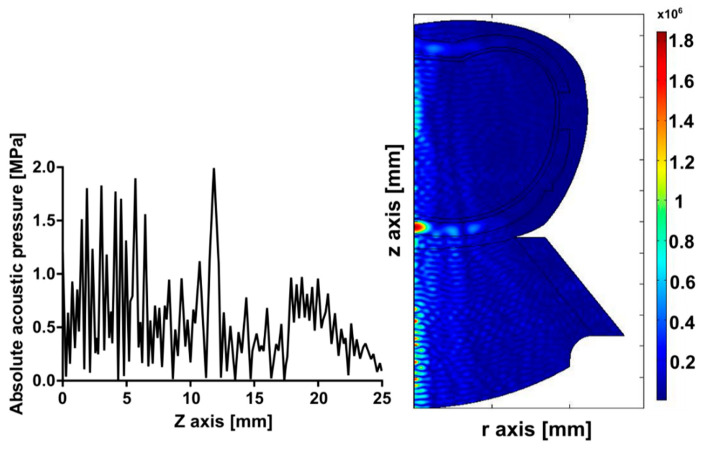
Modeled acoustic pressure. (**Left**) absolute acoustic field along the propagation axis. (**Right**) 2D acoustic field pattern.

To study the effect caused by the skull of the rat, the meninges, and the brain during ultrasound propagation, a study of the acoustic field of the rat head was performed. Figure 3 illustrates how the absorption of acoustic energy deforms the focus and attenuates the acoustic pressure that should reach the brain. This effect was observed during the first animal experiment in vivo.

**Figure 4:**
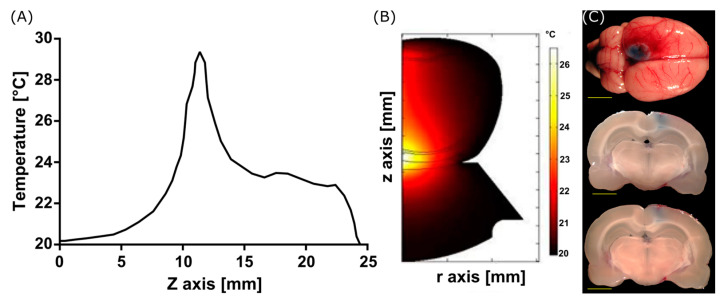
Modeled heating produced by FUS. (**A**) temperature increase through the symmetry axis. (**B**) Heating pattern. (**C**) Complete brain and slices of rats showing the superficial opening of the BBB due to cranium heating (Scale bar, yellow line, is 5 mm).

The models of FUS-induced heating in the rat’s head generate a heating pattern over the tissue on the geometry, as shown in Figure 4. The energy absorption of the bone produces an increase in temperature that could limit the opening of the BBB to the brain cortex of the animal, as shown in Figure 4C. The EB of experiments in vivo reveals a spot at the brain surface, corresponding to the modeled temperature increases shown in Figure 4A,B.

**Figure 5:**
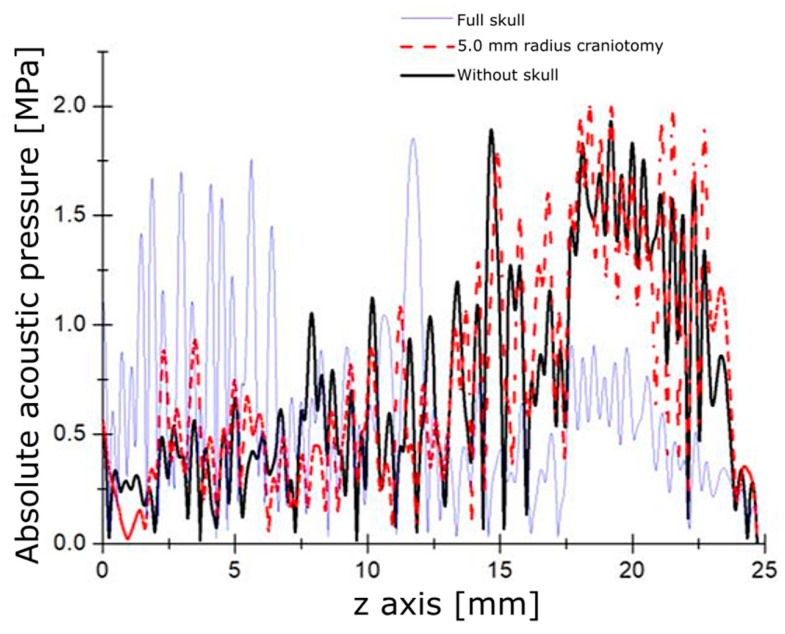
Detailed graph of the acoustic field through 5 mm radius craniotomy.

To avoid this negative effect during the FUS application for opening the BBB, it was proposed performing a craniotomy over the rat’s head. In these simulations, the craniotomies were studied with a different radius of 0.5 mm, 1.5 mm, 3.5 mm, and 5.0 mm. Figure 5 shows that a 5 mm craniotomy allows for the best-case ultrasound propagation in the *z*-axis. The variation between the case with a 5 mm craniotomy and the ideal case with no bone presence was 6%.

**Figure 6:**
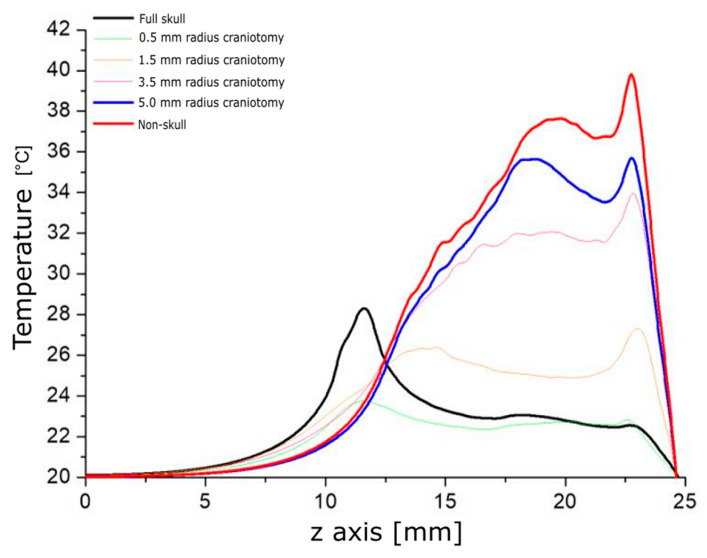
Temperature increases for the analyzed cases.

The heating pattern was studied for every modeled case to analyze the temperature increases, as shown in Figure 6. The craniotomy of a 5 mm radius yielded the best result for heating. This opening ensures that the ultrasound propagation does not deform the focus or the focal length. Additionally, it helps to establish a reference for the characteristics of craniotomy during the in vivo experiments.

### 3.2. BBB Opening with FUS

**Figure 7:**
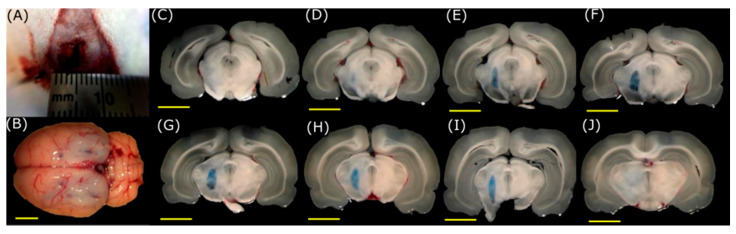
Opening of the BBB using FUS with the transducer targeting the substantia nigra through a square craniotomy of 6 mm per side (equivalent to 3 mm radius in the models). Frame (**A**) corresponds to the craniotomy of 6 mm; a ruler is shown as a scale. Frame (**B**) is a brain extracted from the FUS-treated animal. Frames (**C**–**J**) correspond to serial cuts at the mesencephalon level in the rostral (**C**) to caudal (**J**) direction. It can be noticed that the dye location is only in the FUS-treated substantia nigra (Scale bar, yellow line, is 5 mm).

**Figure 8:**
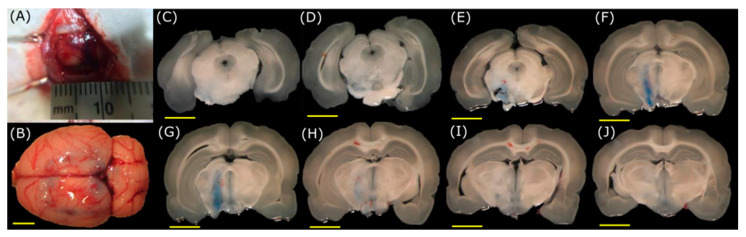
Opening of the BBB using FUS with the transducer targeting the substantia nigra through a square craniotomy of 10 mm per side (equivalent to 5 mm radius in the model). Frame (**A**) corresponds to the craniotomy of 10 mm; a ruler is shown as a scale. Frame (**B**) is a brain extracted from the FUS-treated animal. Frames (**C**–**J**) correspond to serial cuts at the mesencephalon level in the rostral (**C**) to caudal (**J**) direction. It can be noticed that the dye location is only in the FUS-treated substantia nigra (Scale bar, yellow line, is 5 mm).

With the coordinates mentioned above, the axis of the stereotaxic system was adjusted, and the transducer focus was positioned over the substantia nigra. These coordinates were used for all the experiments on animals. Craniotomies were tested and designed with different dimensions according to the models. The presence of EB in different zones of the slices will help us identify if the BBB has been opened. For the craniotomies of 1 mm and 3 mm per side, the temperature increases did not allow the acoustic pressure to reach the focus at the substantia nigra due to the attenuation produced by the skull. The 6 mm and 10 mm craniotomies per side (equivalent to craniotomies of 3 mm and 5 mm radius, respectively), shown in Figure 7 and Figure 8, respectively, provide wider windows for acoustic propagation. These last two cases of windows allowed the temperature to increase enough to open the BBB.

However, in the case of 6 mm square craniotomy (3 mm radius), an interaction occurs between a part of the skull and the ultrasound, which deviates the field profile from the region of interest (Figure 7). The best result was achieved with the 10 mm craniotomy (equivalent to a 5 mm radius in the models) shown in Figure 8 with the EB spots in the region of interest. The created window allows the acoustic field pattern to remain unaffected, which permits adequately reaching the substantia nigra. Additionally, these brain sections show that using FUS to open the BBB did not cause considerable damage to the surrounding brain tissues.

### 3.3. BBB Disruption Reversibility

**Figure 9:**
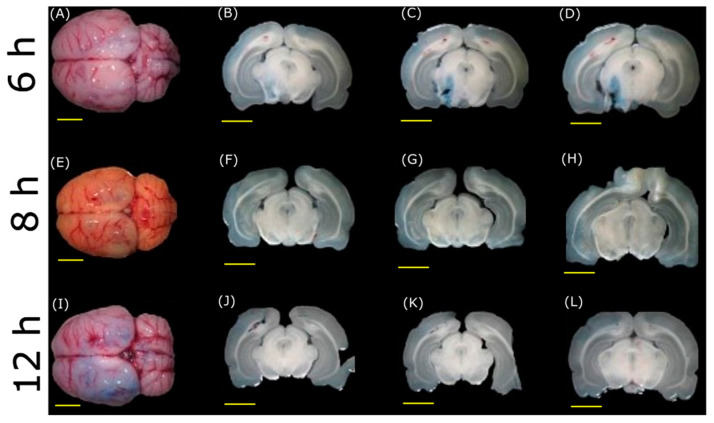
Reversibility corroboration during the experiments of the opening of the BBB. Each column corresponds to different times of EB intravenous injection after FUS application. In the first row, frame (**A**) is the brain of an animal injected with EB at 6 h after FUS application, and the corresponding cuts are shown in frames (**B**–**D**). In the second row, frame (**E**) of an animal injected with EB at 8 h after FUS application, and the corresponding cuts are shown in frames (**F**–**H**). In the third row, frame (**I**) is the brain of an animal injected with EB at 12 h after FUS application, and the corresponding cuts are shown in frames (**J**–**L**) (Scale bar, yellow line, is 5 mm).

To determine the BBB opening, the FUS application and the craniotomies in different rats were performed with EB injections at different hours after sonication. Figure 9 shows brains with the slices made at different times after every procedure. The closing of the BBB occurs after 6 h of FUS application when it is still possible to observe the EB dye in the brain. After 8 h, the BBB is completely closed. These experiments demonstrate that FUS produces a reversible opening in the BBB and highlight the usefulness of ultrasound as a viable tool to propose treatments for neurodegenerative diseases.

## 4. Discussion

This article has been approached with a numerical simulation and in vivo experiments in rats to achieve a protocol for the temporary opening of the Blood–Brain Barrier with focused ultrasound. An experimental setup for the FUS application in male Wistar rats for the opening of the BBB was proposed. The animal preparation was detailed by starting with the weighting of the animal and finishing with the injection of the dye in the bloodstream to verify the opening of the BBB. To identify the temporary opening of the BBB mediated by FUS, a time analysis was performed by injecting Evans Blue several hours after the FUS application. The characteristics of our experimental protocol were obtained with the support of a numerical simulation based on the Finite Element Method in which the acoustic pressure distribution to different craniotomies in a rat model, and the thermal analysis of temperature increases produced by the FUS in soft tissue was analyzed.

Starting the discussion with the numerical simulations by describing the acoustic propagation analysis, in Figure 3, it can be noticed that the bone plays an important role in absorbing the acoustic energy before reaching the interior of the brain. This absorption produces a deformation of the acoustic field at the focus, which impedes the waves from reaching the desired zone. The heat produced under these conditions is shown in Figure 4 with predictable results. These computational findings demonstrate the increased and superficial heat concentration by the presence of bones that consequently leads to undesired effects on the brain tissue. From the experiments in rats shown in Figure 4C, the heat was only located in the more external region of the brain, at the cerebral cortex. The area of interest, the substantia nigra, was not reached at all. Our simulations have considered the 2D anatomic structure of the rat’s head, unlike other work [12] in which the analysis of their models is carried out in 1D.

As mentioned before, bone produces heat, so we carried out the model, considering craniotomies, to avoid absorption by the bone and to facilitate the passage of the ultrasound wave throughout the tissue. Figure 5 shows the acoustic pressure distribution through a 10 mm diameter craniotomy compared with the case with bone and without cranium. By comparing the acoustic pressure transmission, it seems that the graphs are very close in the cases of 10 mm diameter craniotomy and removing all the pieces of the skull. Therefore, just removing a portion of bone instead of the entire skull permits FUS to produce similar effects and reach the desired zone. Performing craniotomies also helps to reduce superficial brain damage by temperature increase at the cranium; other studies have not taken into account this temperature increase [48].

From the results obtained from the numerical simulations, in Figure 6, it can be noticed that craniotomies with the smallest radius (0.5 mm, 1.5 mm) produce a displaced thermal focus close to the cranium with small temperature increases. In the cases of larger craniotomies (3.5 mm, 5.0 mm) or of non-skull, the thermal focus is deeper as the higher temperature increases, with the best results for the 5.0 mm radius craniotomy. While some works proposed the application of craniotomies with the removal of two pieces of bone [13], in this study, it was shown that only a small size of craniotomy is enough to reach the desired zone, i.e., the substantia nigra.

Concerning the experimental results, the effects mentioned before were also observed in animal experiments of Figure 7 and Figure 8, in which the best outcome was achieved with a craniotomy size of 10 mm per side (equivalent to a 5.0 mm radius in the models). This craniotomy size, in conjunction with the size of the acoustic field of the used transducer, permits the passage of the most acoustic energy, ensuring efficient thermal targeting of the desired zone. Although it was possible to reach a deeper region of the brain with a 6 mm square craniotomy (3 mm radius) in animal experiments in Figure 7, it was not possible to predict the exact position of the focus due to the physical interference of the cranium. Conversely, for the results shown in Figure 8 with 10 mm square craniotomy (5 mm radius), the focus was accurately placed at the substantia nigra, as predicted by the models, half of the size from other work groups [21]. Slices with no EB are shown to evaluate the absence of dye in regions outside the ultrasound focus.

Immediately after applying FUS in the animal experiments (Figure 7 and Figure 8), the dye effectively crosses the BBB. Moreover, the EB still crosses the BBB when injecting the dye 6 h after the FUS application, thus indicating the barrier is still open. This is not the case after 8 h of the application of FUS, in which there is no evidence of EB in the brain, as it seems in Figure 9 of the experiments in vivo. Time course experiments demonstrate that the barrier reconstructs itself after being open for about 6 h, i.e., the BBB opens temporarily. This point has significant importance because maintaining a closed barrier in its natural state is necessary to prevent damage caused by other potentially harming molecules. In contrast, other studies have demonstrated that the opening of the BBB can reach times of 24 h [49].

The importance of performing a craniotomy before the FUS application was to generate minimum damage to the soft tissue by temperature increases. Therefore, the numerical simulations performed helped us determine the optimum size of the window to be made in the cranium before applying FUS. If a craniotomy is not performed, two problems arise: the temperature increases in both a zone near the bone and the brain surface and the incapability to position the focus in the desired area of the brain, in our case, the substantia nigra in rats.

The protocol developed in this study can produce the temporary opening of the BBB by 2.5 min of FUS application after a craniotomy and its reversibility to its natural state 6 h after the FUS application. To achieve the opening of the BBB, other protocols require the repetition of the FUS application for several minutes after the first application [19]; even if the FUS application is in short pulses. The prolonged time because of two or more FUS applications requires maintaining the animal sedated for a long time. The use of microbubbles by Todd et al. [50] or Wang et al. [26] has been proposed as an enhancer for the opening of the BBB mediated with FUS; however, our method causes the opening of the BBB without employing microbubbles.

Our results show that the FUS protocol developed to open the BBB in the substantia nigra temporarily and reversibly was efficient in male Wistar rats with negligible secondary effects due to the thermal increase. Therefore, our study supports the use of FUS as a reliable tool that offers advantages over the widely used invasive methods, such as direct needle insertion for drug delivery into the rat brain. The present research is sustained on computational models by FEM that enabled us to predict outcomes from experiments in vivo when the brain is irradiated by ultrasound through different size craniotomies. Finally, experiments with FUS through different craniotomies validated the predictions yielded from the computational models. Preliminary results of our group show that this FUS protocol enables a safe and efficient gene delivery by a synthetic nanovector to dopaminergic neurons of the substantia nigra in Wistar rats. Therefore, this protocol can help implement drug or gene delivery approaches in preclinical studies of PD.

## 5. Conclusions

Based on the obtained results, it is viable to conclude that the designed protocol produces a temporary, localized, and reversible opening of the BBB and can be considered a minimally invasive tool. The sonication protocol opens the BBB in 2.5 min, and the closure of the BBB occurs 6 h after the application of ultrasound. The sizes of craniotomies also play an essential role in reaching the substantia nigra in the rat head. Analysis of the closure of the BBB helped us to determine the time in which the BBB returns to its natural state.

The experimental protocol is supported by the numerical simulations performed, which helped us to determine the parameters of the experimental protocol in vivo. The developed protocol is minimally invasive and helpful for transiently opening the BBB.

## Data Availability

Data is contained within the article.

## Supplementary Materials

The following supporting information can be downloaded at: https://www.mdpi.com/article/10.3390/pharmaceutics15122733/s1, Protocol for the temporary opening of the Blood–Brain Barrier (BBB) with Focused Ultrasound (FUS).
