# Supplementary material for: Protocol to Induce the Temporary Opening of the Blood–Brain Barrier with Short-Time Focused Ultrasound in Rats
Source: Pharmaceutics. 2023 Dec 6;15(12):2733. doi: 10.3390/pharmaceutics15122733 (PMC10748005; doi:10.3390/pharmaceutics15122733)
Supplement: Supplementary file 1 [file pharmaceutics-15-02733-s001.zip › pharmaceutics-2685708-supplementary.pdf]

Protocol for the temporary opening of the Blood-Brain Barrier (BBB) with Focused Ultrasound (FUS).

The objective of this protocol is to produce the temporary opening of the Blood-Brain Barrier in Wistar Male Rats using Focused Ultrasound (FUS) positioning the focus generated over the *substantia nigra*. The in-vivo tests were performed under the protocol 162-15 approved by the ethical committee of CINVESTAV. The animals used during the experimental work were Wistar male rats with weight between 210-230 g (Figure 1). This protocol detailed step by step all the handling and experimental work performed.

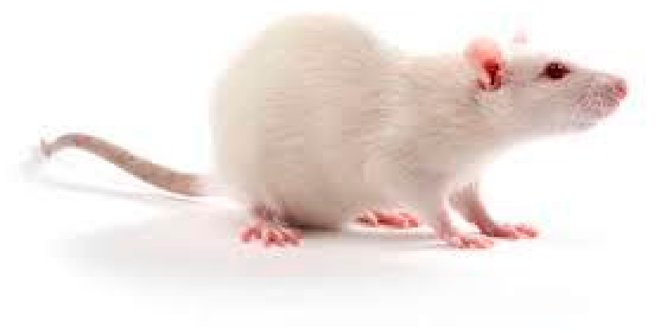

Figure S1. Wistar male rat.

Materials

- Xylazine (PISA agropecuaria, Reg. S.A.G.A.R.P.A. Q-7833-099)
- (PISA agropecuaria, Reg. S.A.G.A.R.P.A. Q-7833-028)
- Electric razor (Wahl, Clipper Pet)
- Stereotaxic system for rats (Stoelting, 51600).
- Lidocaine (Xylocaína, 2 % solution)
- Surgical scalpel and forceps
- Hydrogen peroxide (H2O2)
- Drill (Dremel Multipro, 395)
- 1 mm bits
- Saline solution
- Bidistilled and degasified water
- TPU cone (homemade)
- Acoustic gel
- FUS transducer (Onda Corporation, 2-20-20/4)
- Impedance coupling (Onda Corporation, 2-20-20/1)
- Signal generator (Tektronic, AFG3021B)
- Digital oscilloscope ()
- Power amplifier (Amplifier Research, 500A250)
- Power meter (Amplifier Research, PM2002)
- Bone wax
- Evans Blue (Sigma-Aldrich, Evans Blue)
- Pentobarbital sodium (Pisabental, PISA agropecuaria, Reg. S.A.G.A.R.P.A. Q-7833-215)

- Vibratome (VT1200S, Leica, USA)
- Rat guillotine (Nemi scientific, USA)

Methods

1.- All the animals were applied a composition of anesthesia by 990 µl Xylazine and 2540 µl Ketamine, determined by a relation of 0.32 µl for every 220 g of weight of the animal. The rat is weighed to calculate the dose of anesthetic. This anesthetic is applied intraperitoneal via.

2.- When the animal is under the effect of the anesthetic, it is shaved in the head with an electric razor (Figure 2).

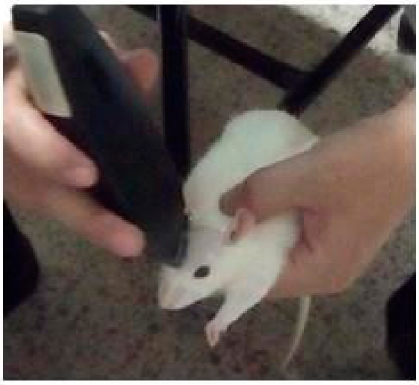

Figure S2. Rat been shaved after anesthesia has been applied.

3.- Once the animal is shaved, it is placed in the stereotaxic system, as it seems in Figure 3. First it is adjusted the head with the lateral bars by inserting the tip in the auditive channels keeping the symmetry in the position. Next, the anterior part of the animal is held supporting the upper incisor teeth in the frontal projection of the stereotaxic system.

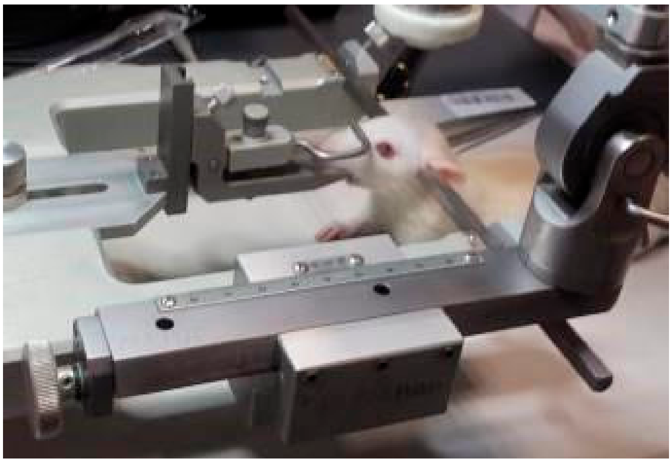

Figure S3. Rat placed in the stereotaxic system.

4.- Prior the incision, it is injected 0.2 ml of lidocaine subcutaneously to decrease the bleeding generated.

5.- For the incision, it is used a scalpel to make a cut from the anterior part of the animal with direction to the posterior part of the animal approximately of 2 cm. To maintain opened the cut performed surgical forceps were used.

6.- Using hydrogen peroxide, it is removed de periosteum from the skull, to allow us a better access to the skull and an improved visualization of the lambda suture.

7.- The next step must be made an adjustment of the coordinate of the stereotaxic system of 2.1 mm in the anteroposterior axis and 2 mm in the transversal axis. This is made with the purpose of positioning the focus of the transducer over the substantia nigra.

8.- Once the position is marked, using a drill and 1 mm drill bits, a craniotomy of 1 cm^2 is performed. The point marked is taken as a center for the craniotomy. Figure 4 shows the procedure of marking and craniotomy.

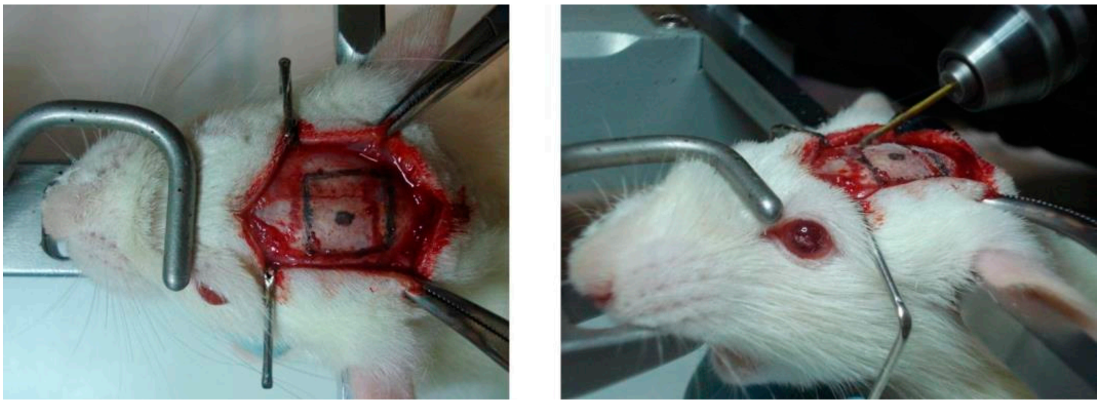

Figure S4. Rat during surgical procedure. To the left is the piece of bone marked to be removed. To the right is during the use of the drill to remove the piece marked previously.

9.- The piece of skull that is cut is removed and placed in saline solution.

10.- We place the transducer over the aperture made.

11.- A TPU cone is placed in the transducer filled with bidistilled and degasified water and fixed in the arm of the stereotaxic system with the coordinates calibrated before. Between the tip of the cone and the rat head it is placed ultrasonic gel for a better coupling.

12.- Then the FUS application is made. The transducer was driven with a sine signal of 1.965 MHz (measured frequency of operation) and an electrical power of 5 W, modulated with a tone burst of 10 Hz; the duty cycle (DC) of the FUS was controlled according to the next sequence:

1. 10 s, DC = 60%,
2. 50 s, DC = 20%,
3. 30 s, DC = 0% (rest),
4. repeat steps 1 and 2.

13.- Once the emission of FUS is finished, the piece of skull previously removed is placed in the rat head and it is secured with bone wax as it shows Figure 5. The cut made is sutured and it is injected 1 ml of Evans Blue by the dorsal vein of the rat tail.

14.- Then, the animal is placed in an acrylic container with a heat source for the recovery of the animal.

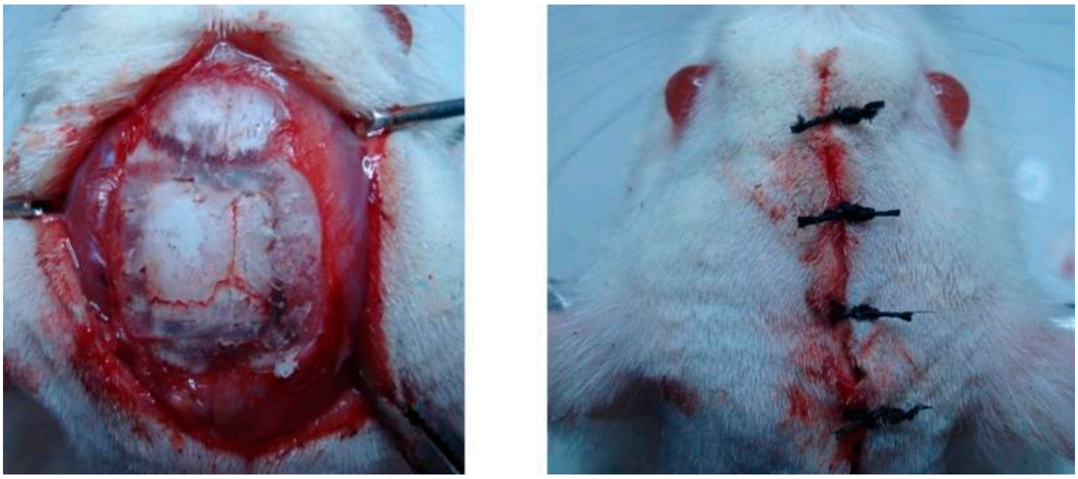

Figure S5. Piece of skull placed and secured in their original spot (left). Suture of the cut previously at the start of the surgery (rigth).

15.- After 24 h of the EB injection, the animal is euthanized. The rat is anesthetized with pentobarbital sodium with a dose of 0.7 ml for every 220 g of weight of the animal. When the rat falls unconscious, is beheaded with a guillotine.

16.- Once the animal is euthanized, the brain is removed from the head.

17.- To maintain the consistency of the tissue and to facilitate cuts, the brain is placed in cold saline solution.

18.- After a few minutes, the cerebellum is removed to place the rest of the brain in the vibratome (VT1200S, Leica, USA) with the posterior face of the brain placed in the base of the vibratome (Figure 6).

19.- The vibratome is turned on and controlled manually to make the cuts to the brain. The slices obtained are 200 µm thickness. These cuts are performed a 1.5 mm/s speed and a displacement of 2 mm.

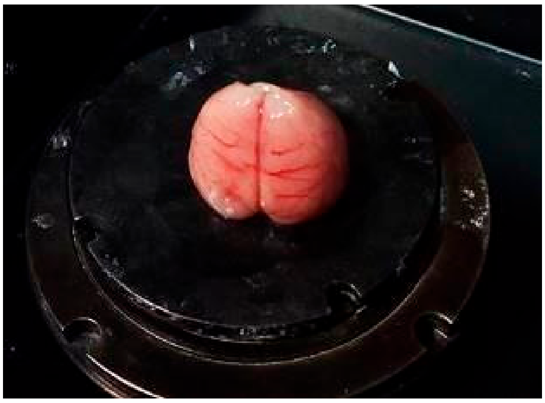

Figure S6. Full brain of the rat place in the vibratome.
